# The role of LINC01419 in regulating the cell stemness in lung adenocarcinoma through recruiting EZH2 and regulating FBP1 expression

**DOI:** 10.1186/s13062-022-00336-8

**Published:** 2022-09-01

**Authors:** Zhao Chen, Weijian Tang, Yuhan Zhou, Zhengfu He

**Affiliations:** Department of Thoracic Surgery, Sir Run Run Shaw Hospital, School of Medicine, Zhejiang University, Qingchun Road East, Hangzhou, 310016 Zhejiang Province China

**Keywords:** LINC01419, EZH2, FBP1, Lung adenocarcinoma, Cancer stem cell

## Abstract

**Background:**

Recent years have witnessed a growing academic interest in the effects of lncRNAs on tumors. LINC01419 is found to facilitate proliferation and metastasis of lung adenocarcinoma (LUAD) cells, but there is a great deal of uncertainty about how LINC01419 works on LUAD cell stemness. For this reason, the focus of this research is centered on the regulatory impact of LINC01419 on LUAD cell stemness.

**Methods:**

For the detection of the expression level of LINC01419 in LUAD, qRT-PCR was performed. And how oe-LINC01419 and sh-LINC01419 affected LUAD cell proliferation as well as stem cell sphere-formation were examined by CCK-8 and cell sphere-forming assays. In addition, whether LINC01419 could recruit EZH2 and regulate FBP1 expression were determined by bioinformatics analysis, RNA immunoprecipitation (RIP), and chromatin immunoprecipitation (ChIP). Western blot was utilized to detect the protein expression levels of FBP1, CD44, CD133, and ALDH-1 as well.

**Results:**

On the basis of the findings from those assays, an up-regulation of LINC01419 level was demonstrated in LUAD cell lines, and a remarkable upregulation of it in CD44 + LUAD cells. In LUAD cells, proliferation and stem cell sphere-formation that were attenuated by LINC01419 knockdown were discovered to be facilitated by LINC01419 overexpression. And a binding relationship between LINC01419 and EZH2 was determined by RIP assay. Besides, EZH2 was capable of binding to FBP1 promoter region, as found by ChIP-PCR assay. Finally, it was demonstrated by in vitro experiments that LINC01419 could inhibit FBP1 expression by recruiting EZH2, resulting in promotion of LUAD cell proliferation and stemness.

**Significance:**

To summarize, our findings demonstrate a cancer-promoting role of LINC01419 in LUAD. LINC01419, by recruiting EZH2 and regulating FBP1 expression, contributes to LUAD cell stemness. According to these findings, the potential of LINC01419 to be the target for LUAD treatment is hence determined, which also adds more possibility to the enrichment of therapeutic strategies for lung cancer stem cells.

**Supplementary Information:**

The online version contains supplementary material available at 10.1186/s13062-022-00336-8.

## Introduction

The presence of cancer stem cells (CSCs) adds more difficulties to the treatment of advanced lung adenocarcinoma (LUAD) [1]. The therapeutic response and prognosis of a variety of cancers can be profoundly affected by CSCs. As stem cells, CSCs carry multiple biological characteristics including self-renewal, replication, undirected differentiation, high tumorigenicity, and resistance to chemoradiotherapy [2]. And those properties of CSCs are of essence during the development of cancers. For instance, the self-renewal and carcinogenesis of colorectal cancer (CRC)-CSCs can be enhanced by KDM2B, propelling malignant progression of CRC [3]. Since there is a close connection between CSCs and the progression of cancers, digging into the underlying mechanisms about how stemness works in LUAD cells is of great significance, which may do a help in finding new therapeutic targets for LUAD treatment.

Extensive studies have mentioned that the involvement of lncRNA in regulating the characteristics of CSCs has an impact on cancer malignant progression. For example, lncRNA DGCR5, by inhibiting the miR-330-5p/CD44 axis, is capable of promoting the stemness of lung CSCs [4]. By activating the Hedgehog signaling pathway, lncRNA-cCSC1 can enhance colorectal CSCs characteristics [5]. Besides, lncRNA MALAT1 facilitates liver CSC characteristics by upregulating YAP1 expression through sponging miR-375 [6]. From abovementioned findings, we can conclude a crucial role of lncRNAs in regulating CSCs properties. In addition, a great deal of research has shown that LINC01419 exerts a role in promoting the proliferation and metastasis of LUAD cells [7]. Nevertheless, the effect of LINC01419 on CSCs is scarcely discussed. Thus, this work looks deeply into the effects of LINC01419 on LUAD stem cells.

Found to be up-regulated in tumors, Enhancer of Zeste 2 Polycomb suppression Complex 2 Subunit (EZH2) is capable of attenuating downstream target gene expression by catalyzing histone H3K27me3 [8, 9]. EZH2 is often recruited to specific genetic loci for silencing the expression of related target genes and exerting its cancer-promoting role [10–12]. In addition, EZH2 is found to be up-regulated in CSCs that can enhance stemness of tumor CSCs [13]. Moreover, it has been studied that some lncRNA functions depend on EZH2 [14, 15]. Abovementioned findings have given us more confidence to investigate whether LINC01419 affects LUAD cell stemness by recruiting EZH2.

As an important regulatory enzyme in gluconeogenesis, Fructose-1,6-bisphosphatase 1 (FBP1) can negatively regulate aerobic glycolysis [16]. In recent years, the cancer-inhibiting role of FBP1 in has been mentioned in various studies. For example, down-regulation of FBP1 facilitates aerobic glycolysis, which in turn promotes proliferation of CRC cells [17]. Fu et al. [18] discovered that breast cancer progression can be propelled by the down-regulation of FBP1 through accelerating the glycolytic process. Additionally, the cellular stemness of CSCs is discovered to be dependent on glycolysis [19, 20]. We next explored the effects of FBP1 on the cell stemness of LUAD cells.

In our research, a remarkable upregulation of LINC01419 was found to exist both in LUAD and LUAD-CSCs cells. LUAD cell proliferation as well as stem cell sphere-formation could be attenuated by the silenced LINC01419. On the contrary, overexpressed LINC01419 had a promotive effect. From a molecular mechanism perspective, LINC01419, by recruiting EZH2, regulates FBP1 expression. The potential of LINC01419 to be a target for the regulation of LUAD stem cells was thus demonstrated.

## Material and methods

### Bioinformatics analysis

LncRNA expression data were downloaded from TCGA-LUAD dataset (59 normal samples, 535 LUAD tissue samples). LINC01419 expression level analysis was performed using TCGA-LUAD dataset. The website (http://pridb.gdcb.iastate.edu/RPISeq/index.html) was utilized to predict a binding relationship between LINC01419 and EZH2. The binding sites of EZH2 on the promoter region of FBP1 were predicted using the hTFtarget database.

### Cell culture and transfection

LUAD cell lines A549 (BNCC337696), HCC78 (BNCC338064), H1975 (BNCC340345), and normal human bronchial epithelial cell line BEAS-2B (BNCC101953) were purchased from BeNa Culture Collection (China). Among them, LUAD cells were cultured using RPMI-1640 medium, and BEAS-2B was cultured using DMEM medium. All the mediums were supplemented with 10% FBS, 100 U/ml penicillin, and 100 μg/ml streptomycin. Then the cells were cultured in a 37 °C constant temperature incubator containing 5% CO_2_.

LUAD-CSCs cell sorting: To investigate the expression of LINC01419 in CSCs, we isolated CD44+ (LUAD stem cells) or CD44− (LUAD non-stem cells) cell population from A549 and HCC78 cell lines using CD44 MicroBead kits (cat. no. 130–095-194, Miltenyi Biotec), which were performed according to the operating instructions provided by the manufacturer [3].

To investigate the effect of abnormal expression of LINC01419, EZH2, and FBP1 on the biological function of LUAD cells, we constructed overexpression vectors oe-LINC01419, oe-EZH2, and the corresponding negative control oe-NC. To silence expression of LINC01419, EZH2, and FBP1, we constructed sh-LINC01419, sh-EZH2, sh-FBP1 and the corresponding negative sh-NC; all sequences in this study were synthesized by Shanghai Sangon (Shanghai, China). LUAD cells were seeded in 6-well plates and cultured to about 60%, and then the above vectors were transfected into the corresponding cells using Lipofectamine 2000 kit, and finally the relevant indicators were detected.

### CCK-8

Transfected LUAD cells were seeded into 96-well culture plates (1 × 10^3^ cells/well). After 0, 24, 48, and 72 h of cell culture, 10 μL of CCK-8 detection reagent was added and the incubation was continued for 2 h. Absorbance values were detected by a microplate reader at 450 nm.

### Cell sphere-forming assay

1 × 10^3^ LUAD cells were seeded in 6-well plates (plates were pretreated with polyHEMA sterile solution) and were placed in standard CSCs medium (DMEM/F12 + 2% B27 + 100 U/ml penicillin + 100 ng/ml streptomycin + 20 ng/ml human-derived EGF + 10 ng/ml human-derived bFGF). 14 days after transfection, and the number of tumor spheres was counted under an inverted microscope.

### RNA immunoprecipitation (RIP)

RIP experiments were performed using an EZMagna RIP kit (Millipore, Billerica, MA, USA) on the basis of the instructions provided by the manufacturer [15]. Briefly, cells were lysed with RIP lysis buffer, and the lysates were incubated with beads bound with antibodies, then the beads were washed with proteinase K to remove proteins, and finally the expression levels were measured by qRT-PCR.

### Chromatin immunoprecipitation (ChIP)

In this study, ChIP experiments were conducted utilizing the Magna ChIP Kit (Millipore, Bedford, MA) under the instruction of the manufacturer [15]. Cells were treated with 4% formaldehyde and the cell lysate was sonicated to interrupt intact chromatin into chromatin fragments (between 200 and 300 bp). Then, chromatin was immunoprecipitated with anti-EZH2 antibody from Abcam, and IgG was treated as a control. The precipitated chromatin DNA was recovered and measured using qRT-PCR. Among them, the primers used for ChIP-PCR are shown in Additional file 2: Table S1.

### qRT-PCR

Total RNA extraction was conducted by using Trizol reagent, which was then reversely transcribed into cDNA using a reverse transcription kit. The SYBR Premix Ex Taq II kit was employed for conducting qRT-PCR**,** and qRT-PCR was operated utilizing Applied Biosystems 7500 Real Time PCR system taking GAPDH as internal reference. The primers used in this study are shown in the Additional file 3: Table S2.

### Western blot assay

Total proteins extraction was performed by using RIPA lysate. BSA kit was utilized for examining total protein concentration. Then protein was separated by SDS-PAGE, and transferred onto PVDF membrane. Next, the membrane was blocked for 1 h and incubated with primary antibodies (EZH2 (ab191250, 1:5000), FBP1 (ab109020, 1:2000), CD44 (ab243894, 1:1000), CD133 (ab222782, 1:2000), ALDH-1 (ab177463, 1:2000), GAPDH (ab181602, 1:10,000), IgG (ab109489, 1:2000)) overnight at 4 °C. After being washed three times, the membrane was added with horseradish peroxidase (HRP) secondary antibody. Then it was incubated overnight at 4 °C. Finally, the brightness of the protein bands was detected [21], and the above antibodies were purchased from Abcam (UK).

### Animal model construction

All animal experiments were performed in strict accordance with the Guide for the Care and Use of Laboratory Animals and were approved by the Medical Laboratory Animal Care Committee of Sir Run Run Shaw Hospital, School of Medicine, Zhejiang University. For xenograft model construction, a total of 10 male BALB/c nude mice (6-week-old, 20–22 g) were purchased, and the mice were randomly divided into 2 groups (n = 5/group): sh-NC group and sh-LINC01419 group. HCC78 cells transfected with sh-NC and HCC78 cells transfected with sh-LINC01419 (cell concentration of 2 × 10^6^ cells/200 μL) were subcutaneously injected into the flanks of the mice, respectively. During the normal feeding period, the subcutaneous tumor growth of mice was observed and recorded every 7 days, and the tumor weight, tumor length (L), and width (W) were measured and recorded. The tumor volume calculation formula was: tumor volume V = (L × W^2^)/2 mm^3^. 28 mice were fed normally. The mice were anesthetized with 2% pentobarbital sodium (50 mg/kg) and sacrificed by cervical dislocation. Subcutaneous tumors were excised and weighed. All mice were dissected and tumor tissues were collected for subsequent experimental analysis [22, 23].

### Immunohistochemistry (IHC)

In this study, immunohistochemical experiments were conducted using the method described in a previous article [24]. The antibodies used in this study were as follows: FBP1, Ki67, and the above antibodies were purchased from Abcam (UK).

### Dual-luciferase assay

The FBP1 promoter region containing the binding site was cloned into pGL3-basic (Panomics, Fremont, CA, USA) and designated as FBP1 WT. All binding site mutated FBP1 promoter regions were cloned into pGL3-basic and designated as FBP1 MT. pRLSV40 (Promega, Fitchburg, WI, USA) was used as a control. To investigate the effect of silencing or overexpression of EZH2 on FBP1 transcriptional activity, sh-EZH2 or oe-EZH2 was co-transferred into LUAD cells and luciferase activity was measured using a dual-luciferase reporter assay system.

### Statistical analysis

All data for this study are presented as mean ± standard deviation. All experiments were repeated at least three times. All data were analyzed using GraphPad Prism 8.0 (CA, USA) and compared between two or more groups using Student’s-t test or ANOVA test. *P* < 0.05 was considered statistically significant.

## Results

### LINC01419 is up-regulated in LUAD

**Fig. 1:**
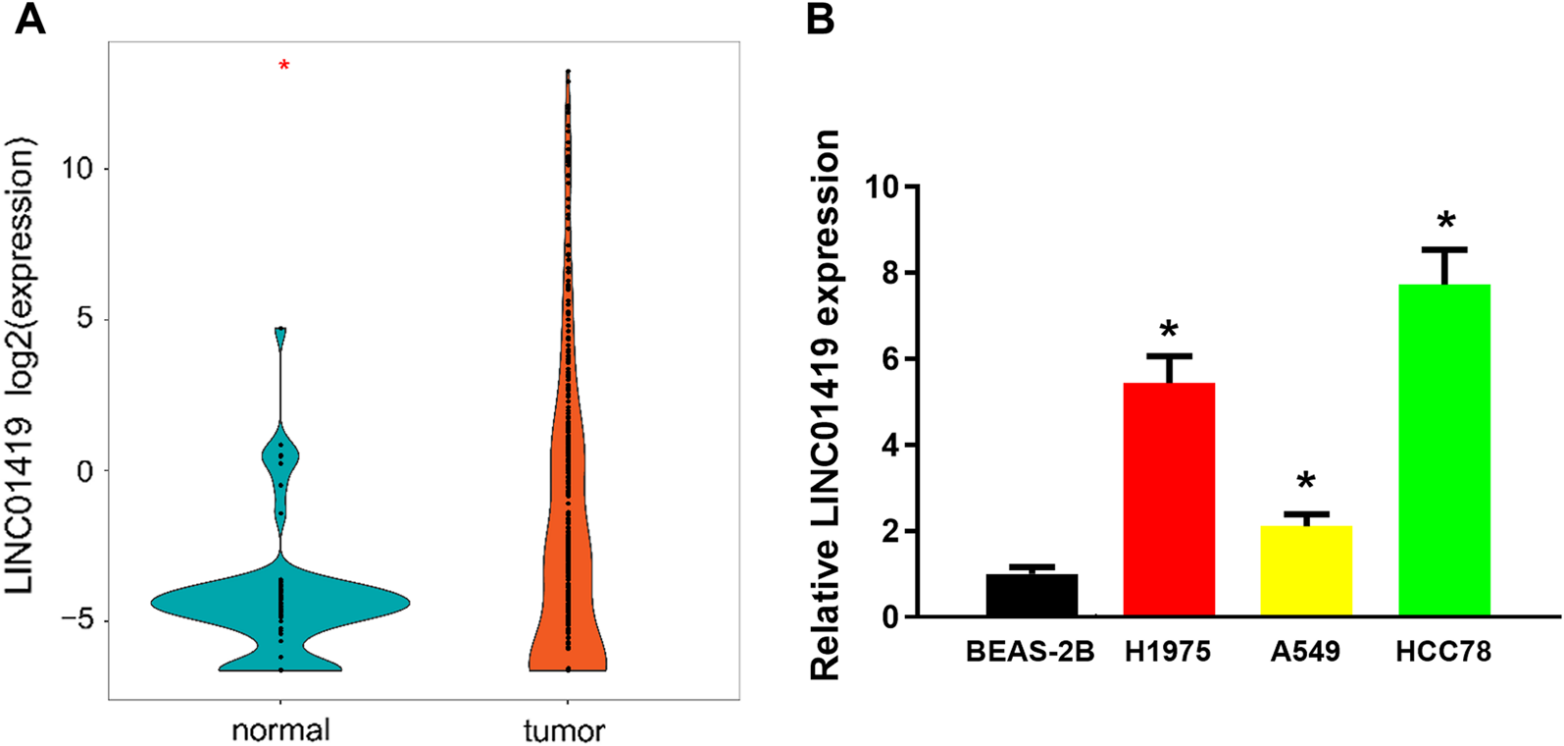
LINC01419 is up-regulated in LUAD. **A** Expression analysis of LINC01419 in TCGA-LUAD dataset. Blue represents normal tissue (n = 59), and orange represents tumor tissue (n = 535); **B** Expression level of LINC01419 in LUAD cell lines and normal human lung cells detected by qRT-PCR; *P < 0.05 indicates statistically significant difference

It has been shown that LINC01419 is up-regulated in LUAD and plays a cancer-promoting role [7]. Therefore, the expression level of LINC01419 in LUAD by TCGA-LUAD database was firstly analyzed, and the result demonstrated that LINC01419 was significantly up-regulated in LUAD patient’s tissues (Fig. 1A). In addition, the expression level of LINC01419 in LUAD cell lines was detected by qRT-PCR, and LINC01419 was significantly up-regulated compared with normal lung cells (Fig. 1B), indicating that LINC01419 may play a cancer-promoting role in LUAD.

### Aberrant LINC01419 expression affects LUAD cell proliferation and cell stemness

**Fig. 2:**
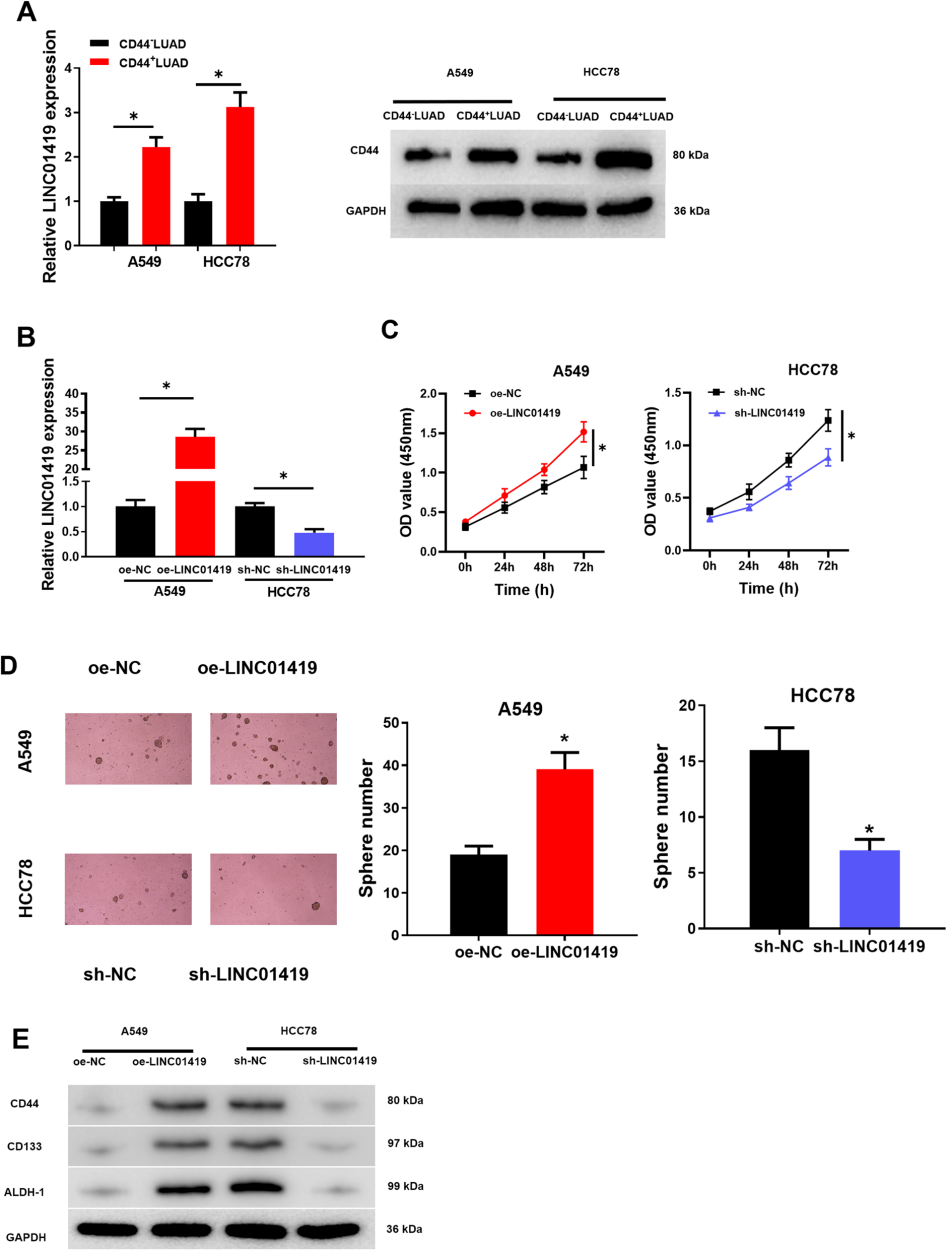
Aberrant LINC01419 expression affects LUAD stem cell properties. **A** qRT-PCR and western blot were used to detect the expression level of LINC01419 and CD44, respectively; **B** qRT-PCR was used to detect the expression level of LINC01419 in oe-LINC01419 cells and sh-LINC01419 cells; **C** CCK-8 was used to detect the cell proliferation viability of oe-LINC01419 cells and sh-LINC01419 cells; **D** Tumor sphere-forming assay was used to detect the sphere-forming ability of LUAD cells; **E** Western blot was used to detect the expression levels of LUAD-CSC surface markers CD44, CD133 and ALDH-1 in the cells of each treatment group; *P < 0.05 indicates significant differences

While it has been demonstrated that LINC01419 can promote LUAD proliferation and metastasis, the role of LINC01419 in LUAD stem cells is unknown. To further investigate whether abnormal LINC01419 expression affects LUAD cell stemness, the expression levels of LINC01419 in CD44 + LUAD and CD44- LUAD cells were detected by qRT-PCR and the expression levels of CD44 in CD44 + LUAD and CD44- LUAD cells were detected by western blot. LINC01419 presented a remarkable upregulation in CD44 + LUAD cells with a contrast to the control group (Fig. 2A), indicating that LINC01419 may be involved in regulating LUAD cell stemness. Subsequently, we constructed the oe-LINC01419 cell line and the sh-LINC01419 cell line. Also, a Pearson analysis between LINC01419 and stemness-associated markers was introduced based on TCGA-LUAD dataset (Additional file 1: Fig. S1). qRT-PCR was employed to examine cell transfection efficiency. LINC01419 showed a marked up-regulation in oe-LINC01419 group, but it showed a considerable down-regulation in sh-LINC01419 group (Fig. 2B). Then, as manifested by CCK-8 result, LUAD cell proliferation was promoted by LINC01419 overexpression and inhibited by LINC01419 silencing (Fig. 2C).

In addition, tumor sphere-forming assay showed that LINC01419 overexpression significantly promoted LUAD stem cell sphere-forming, while LINC01419 silencing expression inhibited LUAD stem cell sphere-forming (Fig. 2D). Moreover, numerous studies have shown that CD44, CD133 and ALDH-1 can be used to identify CSCs [25]. Western blot result showed that CD44, CD133, and ALDH-1 expression was considerably elevated in LUAD cells overexpressing LINC01419 compared with controls. However, the expression of CD44, CD133, and ALDH-1 was remarkably decreased in LUAD cells with silenced LINC01419 (Fig. 2E). To sum up, these results indicated that LINC01419 overexpression enhanced LUAD cell stemness, and LINC01419 silencing expression inhibited LUAD cell stemness.

### LINC01419 recruits EZH2

**Fig. 3:**
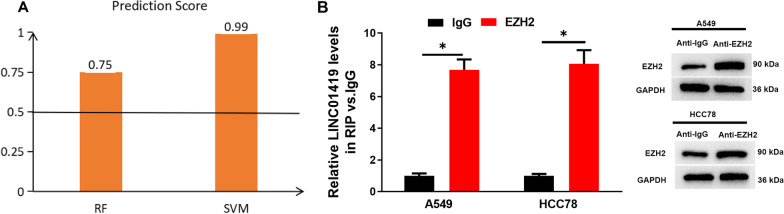
LINC01419 recruits EZH2. **A** RNA–protein interaction predicts the interaction between LINC01419 and EZH2 (score > 0.5 is considered to indicate a binding relationship); **B** RIP and western blot experiments; *P < 0.05 indicates a significant difference

To dive deeper into the mechanism about how LINC01419 regulates stemness in LUAD cells. We found through literature review that the functions of some lncRNAs are dependent on EZH2 [14, 15]. Therefore, we utilized the website (http://pridb.gdcb.iastate.edu/RPISeq/index.html) to predict the relationship between LINC01419 and EZH2. The results of bioinformatics analysis showed that both random forest (RF) and support vector machine (SVM) scores were greater than 0.5 (Fig. 3A). The best value for the score was 1. And the results showed that LINC01419 could recruit EZH2. In the meantime, RIP experiments indicated that LINC01419 bound directly to EZH2. The results of the western blot assay demonstrated that EZH2 showed a markedly higher expression in the Anti-EZH2 group compared with the Anti-IgG group (Fig. 3B). Taken together, these results suggested that LINC01419 recruited EZH2.

### LINC01419 recruits EZH2 to regulate the expression of FBP1

**Fig. 4:**
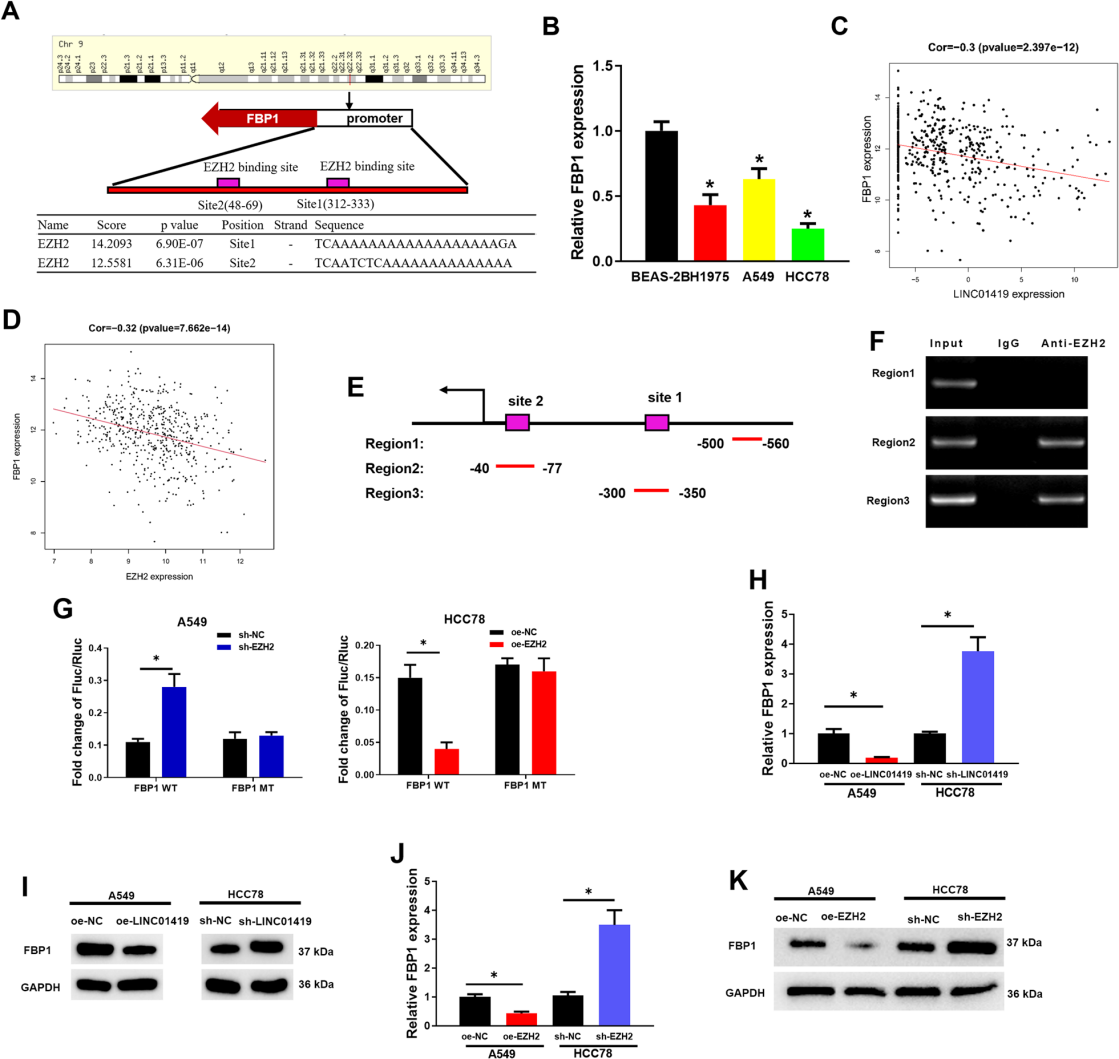
LINC01419 recruits EZH2 to regulate the expression of FBP1. **A** The binding sites of EZH2 to FBP1 were predicted using the hTFtarget database; **B** FBP1 expression level in LUAD cells was measured by qRT-PCR; **C** Pearson correlation between LINC01419 and FBP1 was analyzed; **D** Pearson correlation between EZH2 and FBP1 was analyzed; **E** Schematic diagram of the FBP1 promoter region amplified by different primer sets in ChIP assays, Region2 and Region3 both contained a binding site, and Region 1 without binding site was used as a negative control; **F** ChIP assay was used to detect anti-EZH2 antibody as well as the EZH2 binding PCR products in Region2 and Region3; **G** sh-EZH2 or oe-EZH2 was constructed and co-transfected into LUAD cells with firefly luciferase vectors containing wild-type or mutant FBP1 promoters (FBP1 WT and FBP1 MT); pRL-SV40 was used as a control plasmid containing Renilla luciferase, and the ratio of firefly luciferase to Renilla luciferase was used to determine promoter activity in triplicate for each sample. **H** FBP1 mRNA expression level in LUAD cells transfected with sh-LINC01419 or oe-LINC01419; **I** FBP1 protein level in LUAD cells transfected with sh-LINC01419 or oe-LINC01419; **J**, **K** qRT-PCR and western blot experiments were performed to analyze the expression of FBP1 after silencing and overexpression of EZH2. *P < 0.05 is considered statistically significant

We found a regulatory relationship between LINC01419 and EZH2 in our previous study, and the studies of Xiong et al*.* [26] and Wang et al*.* [27] suggested a possible regulatory relationship between EZH2 and FBP1. Therefore, we chose FBP1 as the target of our study and continued to explore the regulatory mechanisms of LINC01419/EZH2/FBP1 axis in LUAD. To further mine downstream target genes of EZH2, we performed prediction through the hTFtarget database and found that EZH2 had binding sites Site1 (312–333) and Site2 (48–69) on the FBP1 promoter region (Fig. 4A). FBP1 showed a significant down-regulation in LUAD cell lines (Fig. 4B). Pearson correlation analysis showed that LINC01419 demonstrated a remarkably negative correlation with FBP1 expression (Fig. 4C), and EZH2 was also significantly negatively correlated with FBP1 expression (Fig. 4D). Therefore, we designed three pairs of primers in the first 2 kb promoter region of FBP1, in which Region 2 and Region 3 contained EZH2 binding sequences, while Region 1 (without binding elements) serving as a negative control (Fig. 4E). ChIP experiments then showed that distinct bands were observed in Region 2 and Region 3, however no bands appeared in Region 1 (Fig. 4F), indicating a target-binding relationship between EZH2 and the promoter of FBP1.

Subsequently, we further validated the regulatory mechanism of EZH2 binding to FBP1 by dual-luciferase assay. We used an FBP1 promoter-driven luciferase reporter to examine FBP1 transcriptional activity to examine how sh-EZH2 or oe-EZH2 affected luciferase activity. Luciferase activity was significantly increased in sh-EZH2 cells, and it was significantly decreased in oe-EZH2 cells. But sh-EZH2 or oe-EZH2 did not affect the luciferase activity of FBP1 MT (Fig. 4G). It could be seen that EZH2, by directly binding to the FBP1 promoter region, regulated FBP1 expression. Additionally, qRT-PCR and western blot demonstrated that FBP1 was significantly down-regulated in oe-LINC01419 group, while FBP1 demonstrated a remarkable up-regulation in sh-LINC01419 group (Fig. 4H-I). The expression of FBP1 after silencing and overexpression of EZH2 was analyzed by qRT-PCR and western blot assays. It was found that the expression level of FBP1 was significantly down-regulated with EZH2 overexpression and up-regulated with EZH2 silencing (Fig. 4J, K). These results above suggested that LINC01419 may inhibit FBP1 expression by recruiting EZH2.

### LINC01419 affects stem cell characteristics by recruiting EZH2 and regulating FBP1

**Fig. 5:**
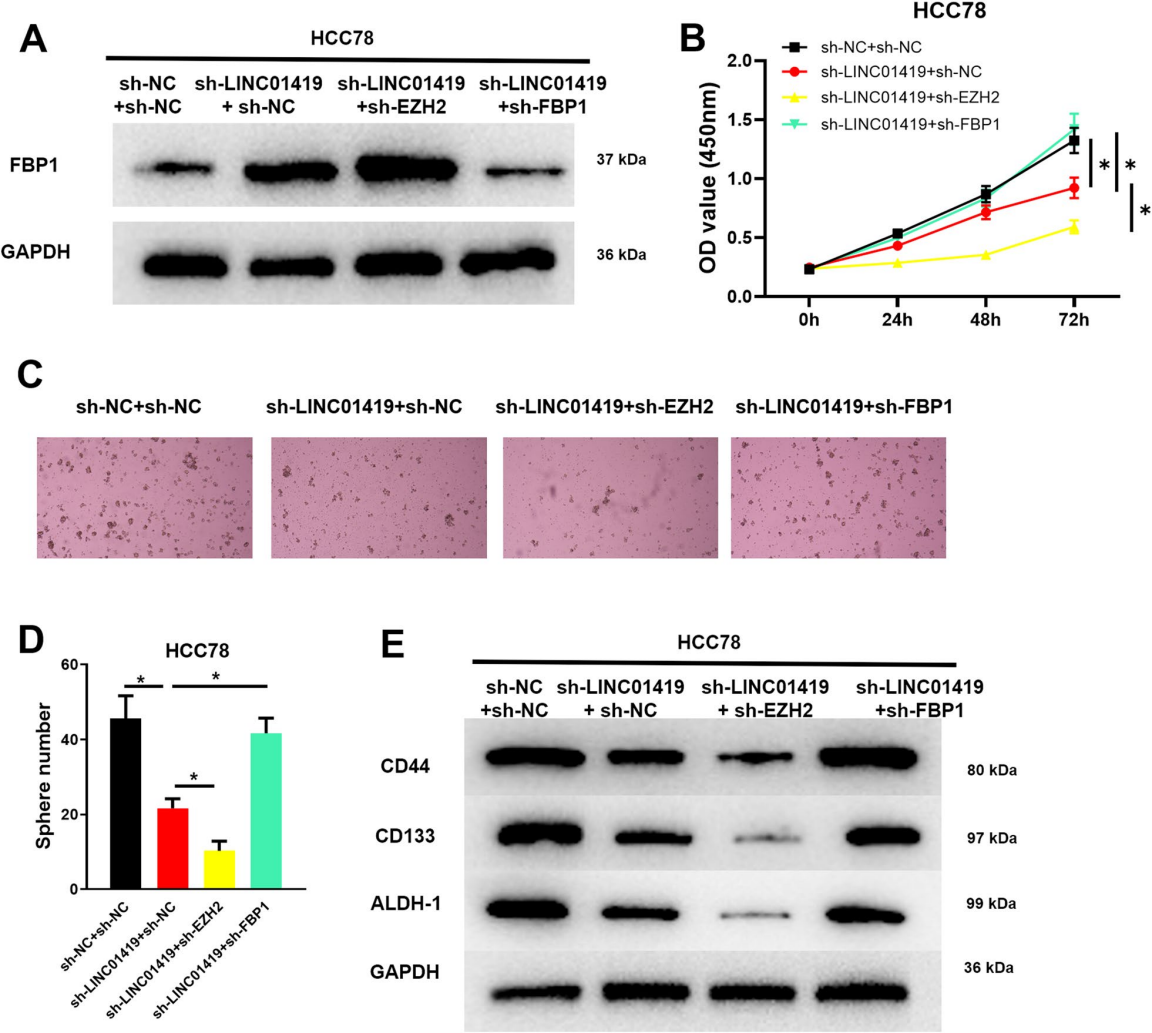
LINC01419 recruits EZH2 to regulate FBP1 affecting the properties of LUAD stem cells (CD44^+^). **A** Western blot was used to detect the protein expression levels of FBP1 in LUAD cells; **B** CCK-8 was used to detect the proliferation activity of LUAD cells; **C** Tumor cell sphere-forming assay was used to detect the sphere-forming ability of LUAD cells; **D** Quantification of the index of the number of tumor spheres in tumor sphere-forming assay; **E** Western blot was used to detect the expression levels of cell surface markers CD44, CD133, and ALDH-1 in each CSCs treatment group. The cell grouping of **A**–**E** was set as follows: sh-NC + sh-NC, sh-LINC01419 + sh-NC, sh-LINC01419 + sh-EZH2 and sh-LINC01419 + sh-FBP1, all of which were transfected into CD44^+^ cells for related studies. *P < 0.05

Since LINC01419 expression was relatively high in HCC78 cells, this cell line was selected for rescue experiments. We constructed sh-LINC01419 and sh-FBP1 vectors to transfect into HCC78 cells and set up the following cell groups: sh-NC + sh-NC, sh-LINC01419 + sh-NC, sh-LINC01419 + sh-EZH2, and sh-LINC01419 + sh-FBP1. First, we examined the cell transfection efficiency of each treatment group using Western blot, and sh-LINC01419 + sh-NC treatment significantly increased FBP1 protein expression. Silencing FBP1 then led to a restore of FBP1 expression level. The expression of FBP1 was significantly upregulated in the sh-LINC01419 + sh-EZH2-treated group compared with the sh-LINC01419 + sh-NC group (Fig. 5A). Then, The cell viability of each treatment group was detected by CCK-8. It was suggested that LINC01419 knockdown significantly hindered LUAD cell proliferation. Silencing EZH2 markedly inhibited proliferation of LUAD cells, and silencing FBP1 restored the proliferative capacity of the cells (Fig. 5B).

In addition, tumor sphere-forming assay showed that LINC01419 silencing expression significantly inhibited LUAD cell sphere-forming, EZH2 silencing expression inhibited LUAD cell sphere-forming. The inhibitory effect of LINC01419 silencing expression on LUAD cell sphere-forming could be reversed by FBP1 knockdown (Fig. 5C, D). The expression levels of cell surface markers in LUAD-CSCs were examined by Western blot, and the expression of CD44, CD133 and ALDH-1 was significantly decreased in LINC01419 silenced expression treatment group compared with the sh-NC + sh-NC group. The expression of CD44, CD133 and ALDH-1 was considerably decreased in EZH2 silenced expression treatment group, and the suppressive effect of LINC01419 silenced expression on CD44, CD133 and ALDH-1 expression could be reversed by FBP1 knockdown (Fig. 5E). These results above indicated that LINC01419 inhibited FBP1 expression by recruiting EZH2, which in turn promoted LUAD-CSCs cell stemness.

### In vivo experiments verifies that LINC01419 silenced expression inhibits LUAD tumor growth

**Fig. 6:**
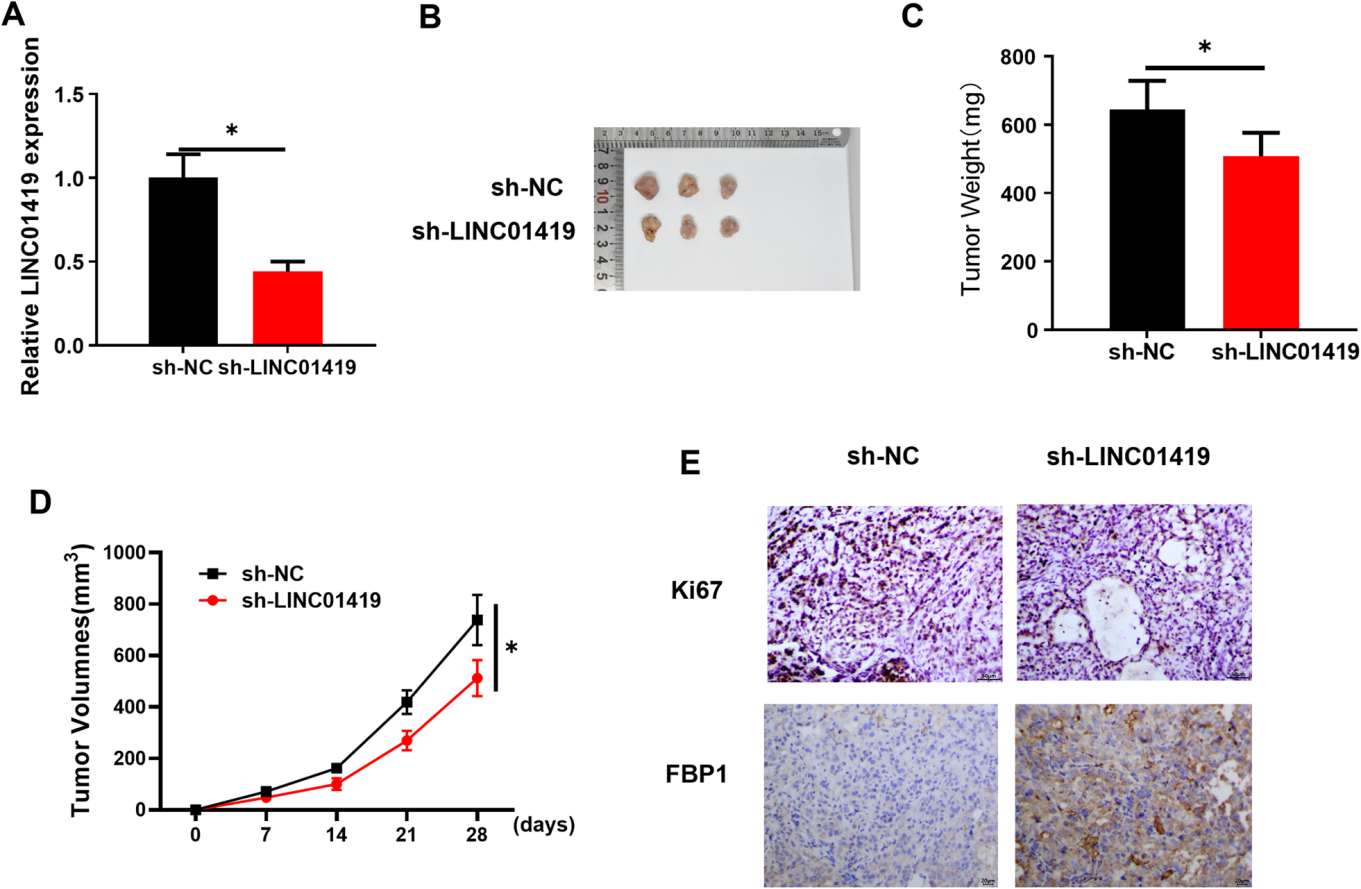
LINC01419 inhibits LUAD tumor growth in vivo. **A** qRT-PCR was used to detect the expression level of LINC01419 tumor tissues; **B** The morphology of xenografts in the two treatment groups of mice (sh-NC and sh-LINC01419 groups); **C** The weight of tumors in the two treatment groups; **D** The growth curve of xenograft; **E** The expression of Ki67 and FBP1 in the xenograft sections was detected by IHC, Scale bars, 20 μm; *P < 0.05 indicates a statistically significant difference

The above experimental data suggested that LINC01419 regulated LUAD cell growth in vitro, but whether LINC01419 could also play a role in vivo was unknown. To explore this, we divided the mice into 2 groups for treatment: (1) sh-NC group, (2) sh-LINC01419 group. First, qRT-PCR result demonstrated a remarkable down-regulation of LINC01419 in tissues after sh-LINC01419 treatment (Fig. 6A). Both tumor weight and volume were markedly decreased in the sh-LINC01419-treated group with a contrast to the sh-NC group (Fig. 6B–D). Meanwhile, IHC assay showed that Ki67 expression was significantly decreased, while FBP1 expression was substantially increased by sh-LINC01419 treatment with a contrast to the sh-NC group (Fig. 6E). Taken together, these experimental results demonstrated that LINC01419 silenced expression hindered LUAD tumor growth in vivo. The *vivo* experiments and in vitro experiments shared a consistent result.

## Discussion

Solid tumors exhibit complex biological characteristics that require a multipronged treatment strategy, and most anticancer treatments (including chemotherapy, radiotherapy) currently fail to prevent tumor resistance and tumor recurrence [28, 29]. Studies have reported that CSCs play a crucial part in the development, progression, therapeutic resistance, and metastasis of tumors [30]. Numerous studies have shown that LUAD-CSCs can activate LUAD tumor formation and recurrence [31, 32]. Therefore, we need to dive into deeper into the molecular regulatory mechanisms that affect the stemness of LUAD cells. This study explored the molecular regulatory mechanism by which LINC01419 enhanced stemness in LUAD cells by recruiting the downstream gene EZH2 and inhibiting FBP1 expression.

LINC01419 is a non-coding RNA longer than 200 bp, and a growing body of studies have shown that LINC01419 is up-regulated in LUAD to play a cancer-promoting role. For example, Cheng et al*.* found that LINC01419 upregulates RCCD1 through sponging miR-519b-3p, and promotes LUAD cell proliferation and metastasis [7]. We further demonstrated that LINC01419 was up-regulated in LUAD by bioinformatics analysis and qRT-PCR assay. Additionally, we found that LINC01419 showed a significant up-regulation in CD44^+^ LUAD cells. For the first time, our study demonstrated the involvement of LINC01419 in the regulation of stemness in LUAD cells. In addition to this, in vitro cell experiments further indicated that LINC01419 overexpression could facilitate CSCs sphere-forming, revealing that LINC01419 could promote the stemness of LUAD cells. In parallel, we utilized known surface molecular markers of CSCs to assess LUAD cell stemness. Researchers have shown that biomarkers such as CD44 [4, 33], CD133, ALDH-1 and Sox2 [31] can be used to identify LUAD-CSCs. In the present study, our experimental results show for the first time that LINC01419 overexpression can promote LUAD cell sphere-formation and promote the expression of stem cell-related markers CD44, CD133 and ALDH-1.

Numerous studies have shown that lncRNAs can regulate the transcription factor EZH2 [14, 15]. EZH2 functions to promote tumor cell proliferation, invasion, and metastasis [34]. Bioinformatics analysis predicted that LINC01419 was able to recruit EZH2. The results of several experiments confirmed the presence of a binding relationship between EZH2 and FBP1. LINC01419 overexpression inhibited FBP1 expression, and knockdown of LINC01419 promoted FBP1 expression. Studies have shown that FBP1 can inhibit the malignant progression of cancers such as hepatocellular carcinoma [35], cholangiocarcinoma [27], and breast cancer [36]. Dong et al*.* showed that FBP1 deletion induces glycolytic processes, promotes EMT pathways in breast cancer cells as well as improves cell stemness in CSCs [36]. Our data indicate that FBP1 silencing expression can enhance LUAD cell proliferation and stem cell sphere-formation, consistent with previous findings.

**Fig. 7:**
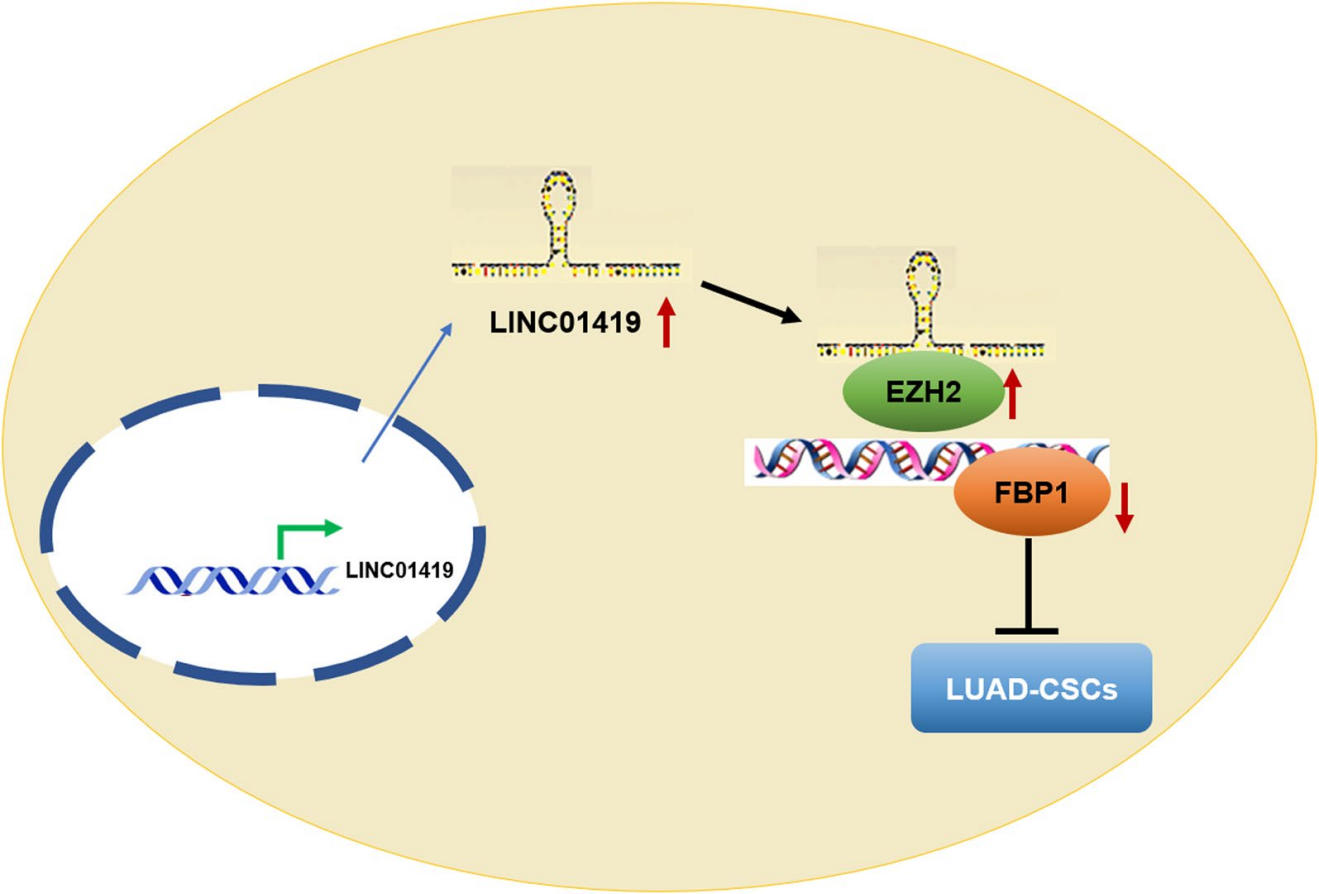
Proposition of a model in which LINC01419 recruits EZH2 and regulates FBP1 to enhance stemness in LUAD cells

In summary, we found through in vitro cell experiments and in vivo experiments that LINC01419 negatively regulated FBP1 expression in LUAD cells, and revealed that the LINC01419/EZH2/FBP1 axis had a pivotal part in LUAD cell proliferation and stem cell sphere formation (Fig. 7). However, there are some shortcomings in our study, for example, the downstream signaling pathways involved in FBP1 are still unclear. Taken together, our study suggests that LINC01419 may be a potential target for LUAD-CSCs therapy, helping to expand ourunderstanding of therapeutic strategies regarding CSCs.

## Supplementary Information

**Additional file 1**: Figure S1. Pearson correlation analysis of LINC01419 with CD44, CD133 and ALDH-1.

**Additional file 2**: Table S1. List of primers used for ChIP-PCR in this study.

**Additional file 3**: Table S2. List of primers used for qRT-PCR in this study.

## Data Availability

The data used to support the findings of this study are included within the article.

## References

[1] Zakaria N (2017). Targeting lung cancer stem cells: research and clinical impacts. Front Oncol.

[2] Li N, Zhu Y (2019). Targeting liver cancer stem cells for the treatment of hepatocellular carcinoma. Therap Adv Gastroenterol.

[3] Sanches JGP (2021). The role of KDM2B and EZH2 in regulating the stemness in colorectal cancer through the PI3K/AKT pathway. Front Oncol.

[4] Wang R, Dong HX, Zeng J, Pan J, Jin XY (2018). LncRNA DGCR5 contributes to CSC-like properties via modulating miR-330-5p/CD44 in NSCLC. J Cell Physiol.

[5] Zhou H (2020). LncRNA-cCSC1 modulates cancer stem cell properties in colorectal cancer via activation of the Hedgehog signaling pathway. J Cell Biochem.

[6] Zhao L, Lou G, Li A, Liu Y (2020). lncRNA MALAT1 modulates cancer stem cell properties of liver cancer cells by regulating YAP1 expression via miR375 sponging. Mol Med Rep.

[7] Cheng Z (2019). LINC01419 promotes cell proliferation and metastasis in lung adenocarcinoma via sponging miR-519b-3p to up-regulate RCCD1. Biochem Biophys Res Commun.

[8] Li Z (2020). Post-translational modifications of EZH2 in cancer. Cell Biosci.

[9] Eich ML, Athar M, Ferguson JE, Varambally S (2020). EZH2-targeted therapies in cancer: hype or a reality. Cancer Res.

[10] Thornton SR, Butty VL, Levine SS, Boyer LA (2014). Polycomb Repressive Complex 2 regulates lineage fidelity during embryonic stem cell differentiation. PLoS ONE.

[11] Lee TI (2006). Control of developmental regulators by Polycomb in human embryonic stem cells. Cell.

[12] Boyer LA (2006). Polycomb complexes repress developmental regulators in murine embryonic stem cells. Nature.

[13] Ezhkova E (2009). Ezh2 orchestrates gene expression for the stepwise differentiation of tissue-specific stem cells. Cell.

[14] Kaneko S (2010). Phosphorylation of the PRC2 component Ezh2 is cell cycle-regulated and up-regulates its binding to ncRNA. Genes Dev.

[15] Lv L (2019). Long non-coding RNA LINC00114 facilitates colorectal cancer development through EZH2/DNMT1-induced miR-133b suppression. Front Oncol.

[16] Yang C (2019). USP44 suppresses pancreatic cancer progression and overcomes gemcitabine resistance by deubiquitinating FBP1. Am J Cancer Res.

[17] Li Q (2019). The FOXC1/FBP1 signaling axis promotes colorectal cancer proliferation by enhancing the Warburg effect. Oncogene.

[18] Fu D (2018). HMGB2 is associated with malignancy and regulates Warburg effect by targeting LDHB and FBP1 in breast cancer. Cell Commun Signal.

[19] Yasuda T, Ishimoto T, Baba H (2021). Conflicting metabolic alterations in cancer stem cells and regulation by the stromal niche. Regen Ther.

[20] Peng F (2018). Glycolysis gatekeeper PDK1 reprograms breast cancer stem cells under hypoxia. Oncogene.

[21] Wang M (2018). Long non-coding RNA H19 confers 5-Fu resistance in colorectal cancer by promoting SIRT1-mediated autophagy. Cell Death Dis.

[22] Zhu Y (2020). LINC00968 can inhibit the progression of lung adenocarcinoma through the miR-21-5p/SMAD7 signal axis. Aging (Albany NY).

[23] Wu J, Sun L, Liu T, Dong G (2021). Ultrasound-targeted microbubble destruction-mediated downregulation of EZH2 inhibits stemness and epithelial-mesenchymal transition of liver cancer stem cells. Onco Targets Ther.

[24] Deng X (2021). A KLF4/PiHL/EZH2/HMGA2 regulatory axis and its function in promoting oxaliplatin-resistance of colorectal cancer. Cell Death Dis.

[25] Li W (2017). Unraveling the roles of CD44/CD24 and ALDH1 as cancer stem cell markers in tumorigenesis and metastasis. Sci Rep.

[26] Xiong X (2020). GSK343 induces programmed cell death through the inhibition of EZH2 and FBP1 in osteosarcoma cells. Cancer Biol Ther.

[27] Wang N (2019). Long noncoding RNA DANCR regulates proliferation and migration by epigenetically silencing FBP1 in tumorigenesis of cholangiocarcinoma. Cell Death Dis.

[28] Dzobo K, Ganz C, Thomford NE, Senthebane DA (2021). Cancer stem cell markers in relation to patient survival outcomes: lessons for integrative diagnostics and next-generation anticancer drug development. OMICS.

[29] Wang X (2018). miR-181b/Notch2 overcome chemoresistance by regulating cancer stem cell-like properties in NSCLC. Stem Cell Res Ther.

[30] Qiu Y, Yang L, Liu H, Luo X (2021). Cancer stem cell-targeted therapeutic approaches for overcoming trastuzumab resistance in HER2-positive breast cancer. Stem Cells.

[31] Chen J, Chen H, Yang H, Dai H (2018). SPC25 upregulation increases cancer stem cell properties in non-small cell lung adenocarcinoma cells and independently predicts poor survival. Biomed Pharmacother.

[32] Zhao S, Shen W, Yu J, Wang L (2018). TBX21 predicts prognosis of patients and drives cancer stem cell maintenance via the TBX21-IL-4 pathway in lung adenocarcinoma. Stem Cell Res Ther.

[33] Herreros-Pomares A (2019). Lung tumorspheres reveal cancer stem cell-like properties and a score with prognostic impact in resected non-small-cell lung cancer. Cell Death Dis.

[34] Yoo KH, Hennighausen L (2012). EZH2 methyltransferase and H3K27 methylation in breast cancer. Int J Biol Sci.

[35] Yang LN, Ning ZY, Wang L, Yan X, Meng ZQ (2019). HSF2 regulates aerobic glycolysis by suppression of FBP1 in hepatocellular carcinoma. Am J Cancer Res.

[36] Dong C (2013). Loss of FBP1 by Snail-mediated repression provides metabolic advantages in basal-like breast cancer. Cancer Cell.

