# Supplementary figures and images for: The role of LINC01419 in regulating the cell stemness in lung adenocarcinoma through recruiting EZH2 and regulating FBP1 expression
Source: Biol Direct. 2022 Sep 1;17:23. doi: 10.1186/s13062-022-00336-8 (PMC9438337; doi:10.1186/s13062-022-00336-8)

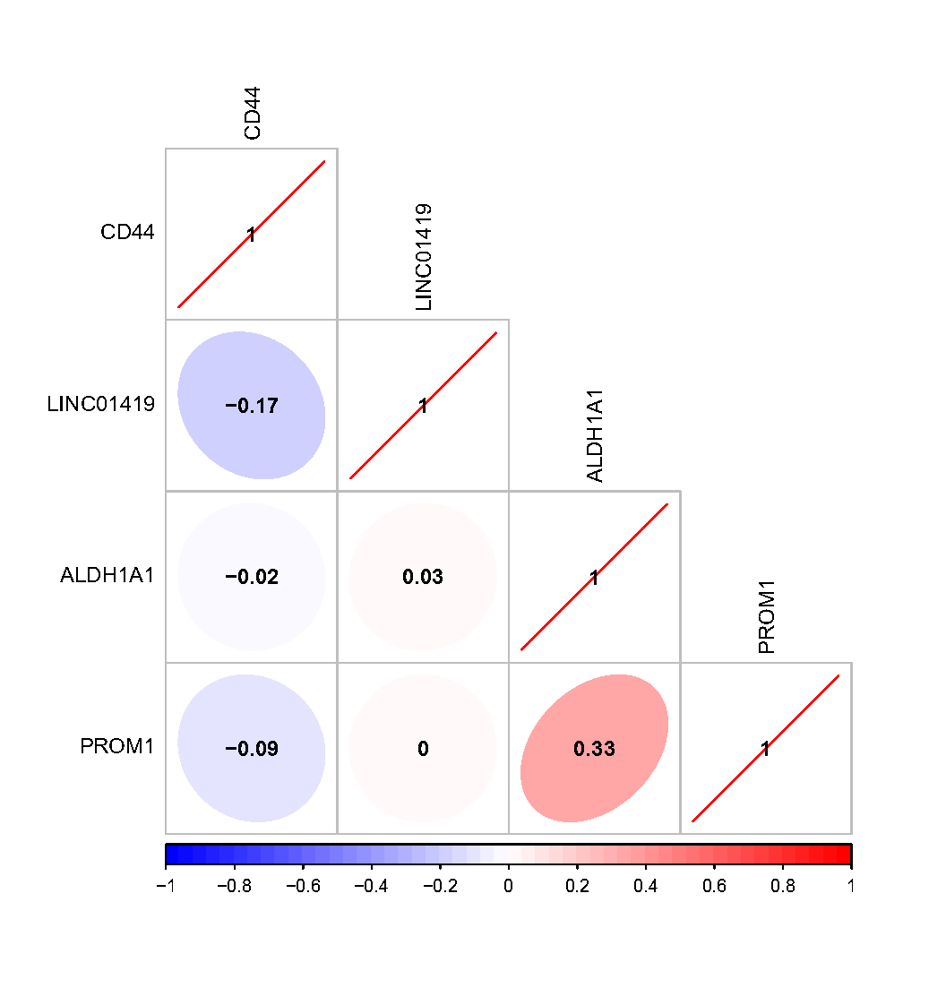

Supplement: Supplementary file 1 — Additional file 1: Figure S1. Pearson correlation analysis of LINC01419 with CD44, CD133 and ALDH-1. [file 13062_2022_336_MOESM1_ESM.tif]
